# Massive Ureterolithiasis

**DOI:** 10.7759/cureus.27234

**Published:** 2022-07-25

**Authors:** Safwan Zaman, Rohan Mangal, Thor S Stead, Jesse Dubey, Latha Ganti

**Affiliations:** 1 Biology, Trinity Preparatory School, Winter Park, USA; 2 Medicine, University of Miami Miller School of Medicine, Miami, USA; 3 Medicine, Warren Alpert Medical School of Brown University, Providence, USA; 4 Emergency Medicine, Lakeland Regional Health, Lakeland, USA; 5 Emergency Medicine, HCA Florida Ocala Hospital, Ocala, USA; 6 Emergency Medicine, Envision Physician Services, Plantation, USA; 7 Emergency Medicine, University of Central Florida College of Medicine, Orlando, USA

**Keywords:** passing, kidney stones, emergency medicine, uterus, massive ureterolithiasis

## Abstract

The authors present the case of a 40-year-old male who visited the emergency department with left-sided flank pain. He was found to have a 12 mm ureteropelvic stone and was provided parenteral analgesia before being admitted to the hospital for urology consultation. The presentation and diagnosis of his case along with treatment options against a backdrop of related studies are discussed.

## Introduction

Renal calculi (kidney stones) are hard deposits of minerals and salts that cluster within the kidneys. Patients with symptomatic nephrolithiasis typically experience severe flank pain that may be associated with hematuria, nausea, vomiting, fever, and chills. The severity of these symptoms increases proportionally with the size of the calculus due to distention of the ureter. Calcium stones are the most common, comprising 80% of renal calculi. Calcium oxalate (CaOx) and calcium phosphate (CaP) or a combination of both constitute the bulk of these renal calculi, and their formation is dependent on urinary pH. A pH between 5.0 and 6.5 favors CaOx, whereas a urinary pH greater than 7.5 is more likely to yield CaP stones. Calcium stones are linked with the greatest recurrence [1]. Uric acid accounts for 10% of all stone types, followed by cystine stones at 2-3%. Certain groups may be at an increased risk of kidney stones. Men have kidney stones at two to three times higher prevalence compared to females [2]. Similarly, men with higher levels of serum testosterone have been linked to a greater risk of kidney stone formation [3]. The size of the calculus is inversely correlated to the spontaneous passage of the stone. A retrospective study of 392 patients demonstrated stones 0-3 mm passing 98% of the time, 81% for 4 mm stones, 65% for 5 mm stones, 33% for 6 mm stones, and only 9% for stones >6.5 mm ion width [4]. Emergency department management consists of hydration and analgesia with non-steroidal anti-inflammatory agents [5]. Obstructive calculi can result in urethral stricture and renal deterioration [6]. This case involves a patient with an abnormally large stone. In cases with kidney calculi less than 5 mm with low chances of passage, shockwave lithotripsy (SWL), ureteroscopy, and percutaneous nephrolithotomy (PCNL) are procedures that may be considered to remove or break the stone into smaller fragments [7].

## Case presentation

**Figure 1:**
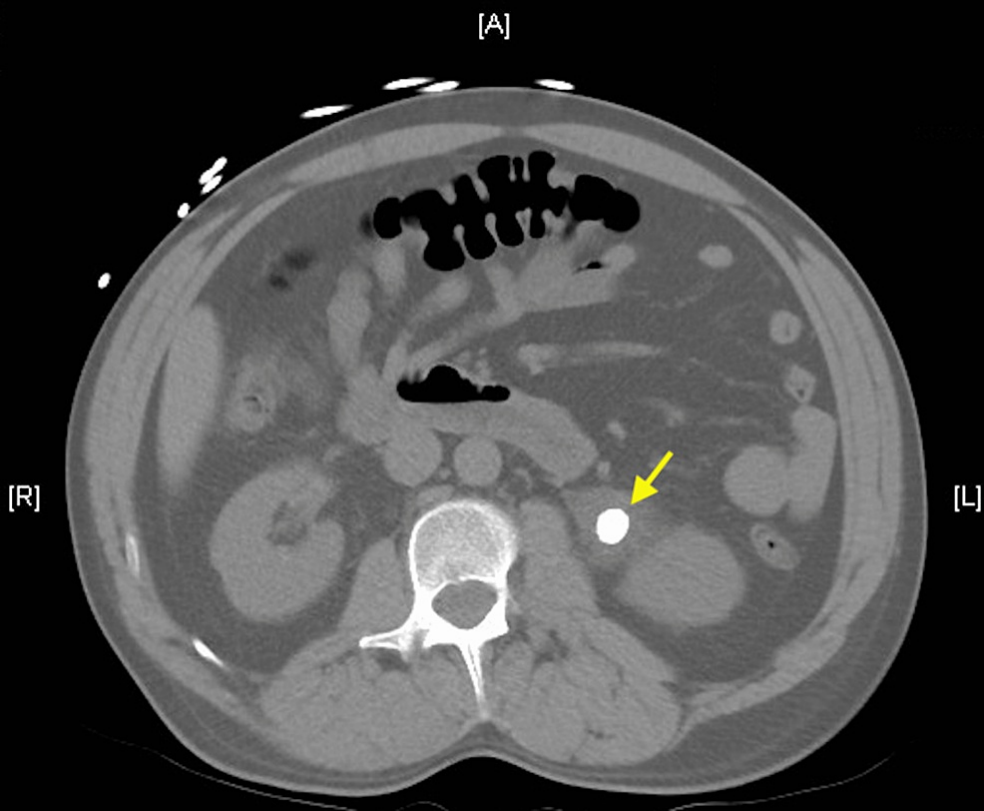
Computed tomography scan visualizing a 12 mm kidney stone in the ureteropelvic junction.

A 40-year-old man arrived at the emergency department with left-sided flank pain, which had begun one day earlier. The pain was described as throbbing and radiated to his left upper quadrant. His vital signs were as follows: temperature of 98.3°F, a pulse of 86 beats per minute, a respiratory rate of 18 breaths per minute, a blood pressure of 168/113 mmHg, and oxygen saturation of 94% on room air. His medical history was significant for nephrolithiasis one year prior. The presentation was similar. At that time, it was an 18 mm stone that required a nephrostomy tube for resolution. His other medical history included chronic low back pain. His surgical history was only significant for myringotomy tubes as a child. He denied any recreational drug use. He was a non-smoker. He did drink alcohol, two beers per day. He worked as a driver. The most prominent finding on physical examination was the exquisite left costovertebral angle tenderness. Examination revealed a well-developed, well-nourished man in moderate distress secondary to pain. Heart, lung, and neurologic examinations were normal. A non-contrast computed tomography (CT) scan revealed a 12 mm stone in the left upper ureteropelvic junction. The patient was treated with 4 mg morphine, 15 mg ketorolac, 2 L intravenous saline solution, and 4 mg ondansetron. He was admitted to the hospital for a urology consultation for the removal of the stone (Figure 1).

Laboratory evaluation was essentially unremarkable; the results are shown in Table 1.

**Table 1:** Laboratory results of the patient.

Parameter	Finding	Normal range
Creatinine	1.10	0.6–1.3 mg/dL
Glucose	115	74–106 mg/dL
Calcium	9.1	8.4–10.1 mg/dL
Calcium adjusted for albumin	9.3	8.8–10.5 mg/dL
Total bilirubin	1.1	0.2–1.5 mg/dL
Aspartate transaminase	35	10–37 unit/L
Alanine transaminase	78	12–78 unit/L
Total alkaline phosphatase	123	45–117 unit/L
Total protein	7.6	6.4–8.2 g/dL
Albumin	3.8	3.4–5.0 g/dL
Blood urea nitrogen/Creatinine ratio	6	
Estimated glomerular filtration rate mL/min		>60

## Discussion

In the management of uncomplicated urolithiasis, the first approach is to evaluate whether the patient is likely to pass the stone on their own. In general, 75-90% of all stones pass without intervention [8]. In this case, the patient had a 12 mm stone. Therefore, regardless of its location, the overall likelihood of this stone spontaneously passing was very low.

There are options for treating large kidney stones that are unlikely to pass. SWL is a noninvasive technique that uses high-intensity pulses to fractionate stones into fragments. These pieces will ideally be small enough to urinate out. Another method is PCNL, which involves surgical removal of the stone. An algorithmic approach can be adopted, depending on the size and location. For lower pole stones <1 cm, either SWL or ureteroscopic retrieval can be employed. Stones measuring 1-1.5 cm likely would need the latter. For stones measuring >1.5 cm, PCNL would be indicated. For upper pole stones, the preferred strategy is usually PCNL [9].

A study examining 383 patients, in which 221 underwent SWL and 144 had PCNL, compared outcomes in kidney stones measuring between 1 cm and 2 cm. While 94% of PCNL candidates were successful, only 76% of SWL patients had success. Although PCNL was the more effective technique in this study, it is the more invasive option as well [10,11]. This decision should be visited once the stone width and location suggest that spontaneous passage is unlikely.

## Conclusions

Renal calculi can be very painful and require immediate attention. Those that are greater than 6 mm in width, regardless of location, are much less likely to be passed without intervention. In this case, the patient had a 12 mm stone that required urologic consultation. While PCNL is a more effective surgical option to remove the stone, SWL should be considered due to its noninvasive nature. We advise that stone location and width should be evaluated before deciding if PCNL or SWL is necessary and which option is the most suitable for the patient.
